# G-quadruplex DNA and ligand interaction in living cells using NMR spectroscopy†
†Electronic supplementary information (ESI) available. See DOI: 10.1039/c4sc03853c



**DOI:** 10.1039/c4sc03853c

**Published:** 2015-01-14

**Authors:** Gilmar F. Salgado, Christian Cazenave, Abdelaziz Kerkour, Jean-Louis Mergny

**Affiliations:** a Univ. Bordeaux , ARNA Laboratory , F-33000 Bordeaux , France . Email: g.salgado@iecb.u-bordeaux.fr ; Fax: +33-5-40-00-30-04; b INSERM , U869 , IECB , F-33600 Pessac , France; c CNRS , Microbiologie Fondamentale et Pathogénicité , UMR 5234 , F-33000 Bordeaux , France; d Univ. Bordeaux , Microbiologie Fondamentale et Pathogénicité , UMR 5234 , F-33000 Bordeaux , France

## Abstract

Gathering structural information from biologically relevant molecules inside living cells has always been a challenging task. In this work, we have used multidimensional NMR spectroscopy to probe DNA G-quadruplexes inside living *Xenopus laevis* oocytes. Some of these structures can be found in key regions of chromosomes. G-quadruplexes are considered potential anticancer therapeutic targets and several lines of evidence indirectly point out roles in key biological processes, such as cell proliferation, genomic instability or replication initiation. However, direct demonstrations of the existence of G-quadruplexes *in vivo* are scarce. Using SOFAST-HMQC type spectra, we probed a tetramolecular G-quadruplex model made of d(TG_4_T)_4_ inside living *Xenopus laevis* oocytes. Our observations lead us to conclude that the quadruplex structure is formed within the cell and that the intracellular environment preferentially selects a conformation that most resembles the one found *in vitro* under KCl conditions. We also show for the first time that specific ligands targeting G-quadruplexes can be studied using high resolution NMR directly inside living cells, opening new avenues to study ligand binding discrimination under physiologically relevant conditions with atomic detail.

## Abstract 2

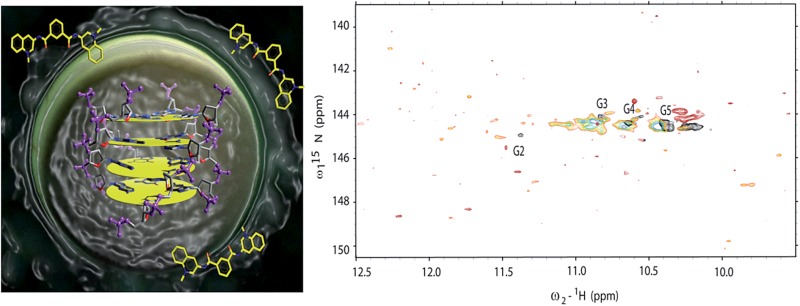
Using in-cell NMR spectroscopy to probe ligand binding to a G-quadruplex nucleic acid.

## Introduction

DNA has been an important target for the development of cancer-related therapies.^1–5^ One of the major problems that arise when choosing DNA as a target is the lack of selectivity for certain gene sequences and cell types, such as tumour cells. An attractive strategy involves finding unique DNA motifs that present important differences in comparison to double helical DNA, as shown initially for DNA triple-helices. G-quadruplex structures fit perfectly in this category.^4^ Recently, new drugs targeting G-quadruplex structures have been developed with anticancer properties, such as quarfloxin.^6^ G-quadruplexes are unusual nucleic acid structures made of stacked guanine quartets. They are stabilized by an assembly of Hoogsteen hydrogen-bonded imino protons across the guanine bases, allowing a diversity of unique structures directly depending on the formation of planar quartets of guanines,^7^ as opposed to the complementary base pairing found in double helical DNA. The resulting structure presents some unique 3D features. The imino signature correlates with the global structural pattern of the G-quadruplex and is very sensitive to different salt types and binding partner species. Interestingly and contrary to a number of other nucleic acid structures, G-quadruplexes are stable under physiological conditions. Usually, two to five quartets of guanines are stacked and further stabilized by monovalent cations, such as K^+^ and Na^+^.^7^ G-quadruplexes can adopt a larger variety of structures and folding features apart from those found in canonical DNA, as reported by numerous studies under *in vitro* conditions.^8–12^ Despite the relatively large amount of information available concerning G-quadruplexes, obtained especially in the last decade, their observation and characterization *in vivo* is rather limited. A few exceptions have emerged recently: using G-quadruplex structure-specific antibodies, Balasubramanian and colleagues were able to study DNA and RNA G-quadruplexes in human cells and observe a structure–function relationship.^13,14^ Another recent report describes the use of monoclonal antibodies that display stronger staining in the presence of G-quadruplex selective ligands inside mammalian cells.^15^

Additionally, recent advances in the NMR field have extended our understanding of how molecules behave inside living cells,^16–19^ allowing the development of innovative ways of studying biomolecules with atomic detail in a physiologically relevant environment compared to *in vitro* studies. Previous studies have attempted to probe the general fold of G-quadruplexes inside cells, and despite the poor resolution the authors were able to infer that the in-cell spectra contained important differences when compared with the *in vitro* spectra.^8,20^ These were important steps in the difficult task of studying biomolecules inside living cells using NMR spectroscopy. In addition, some recent studies point out that molecular crowding conditions may influence some ligand binding properties of G-quadruplexes.^21,22^ In this work we went further by probing the G-quadruplex fold inside living *Xenopus laevis* oocytes in the presence of specific ligands. Using [U-98% ^15^N; U-^13^C]-d(TG_4_T)_4_ oligonucleotides, from now on referred to just as d(TG_4_T)_4_, micro-injected inside living oocytes, we show that G-quadruplexes exist in a folded state inside living cells for relatively long periods of time. Furthermore, when the cells were exposed to media containing a G-quadruplex-selective ligand it was possible to follow the structural modifications in the imino-signature region using NMR spectroscopy. This opens up the possibility for testing ligand affinity and discrimination inside living cells, with the aim to design and improve ligands that could target G-quadruplex motifs or to investigate the conformation of G-rich aptamers within cells. For this study, we have selected d(TG_4_T)_4_ because it is a well-characterized all-parallel-tetramolecular G-quadruplex model, thoroughly studied in the presence of different counter ions and ligands, and by different methods such as mass spectroscopy, circular dichroism, NMR, and X-ray crystallography. Another advantage of using d(TG_4_T)_4_ is that the structure is not concentration dependent. Other tetramolecular all-parallel-stranded G-quadruplexes exist, such as those formed by the oligonucleotide d(^5′^TGGGAG^3′^)^23^ carrying bulky aromatic groups at the 5′ end, which has strong anti-HIV properties. The aptamer *93del*, a potent nanomolar inhibitor of HIV-1 integrase,^24^ also displays all-parallel G-stretches, making this class of quadruplexes biologically relevant.

## Experimental section

### Sample preparation

**Fig. 1:**
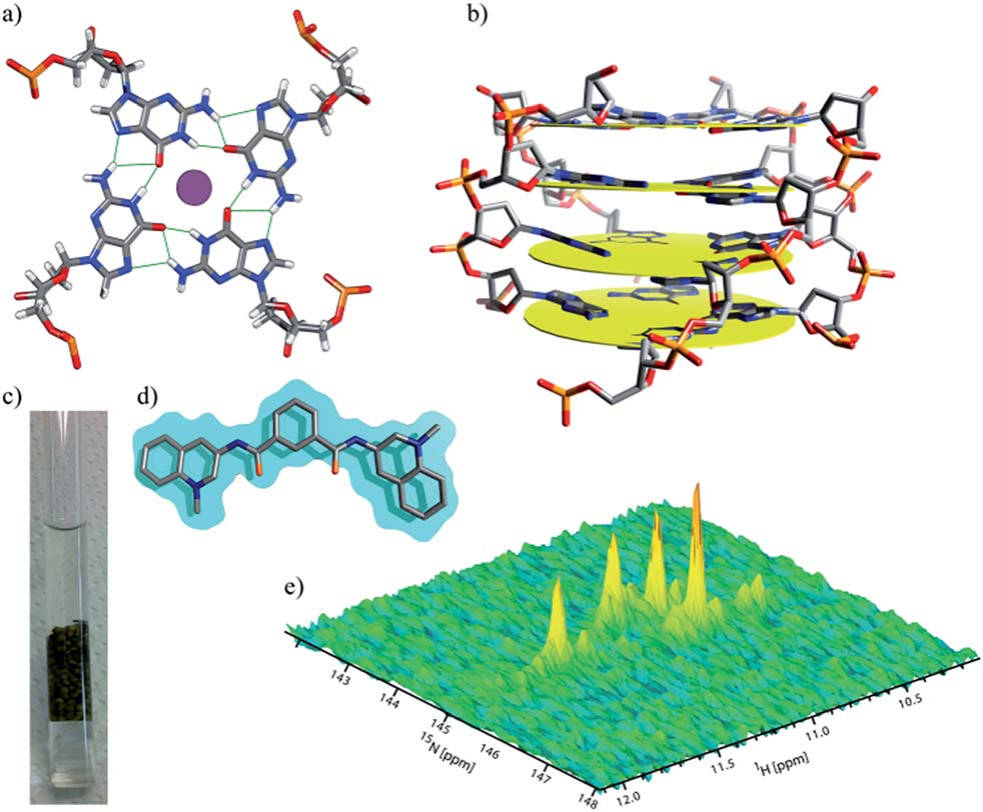
Display of a typical tetrad of guanines, held by a network of hydrogen bonds, coordinating with a central metal cation in between two tetrads (a). Schematic representation of a stack of four tetrads commonly found in d(TG_4_T)_4_ (b). A 5 mm NMR Shigemi tube loaded with ∼200 *Xenopus* oocytes in 20% Ficoll buffer (c). Structure of ligand 360A (d). Imino signature of d(TG_4_T)_4_ in KCl buffer (e) obtained using a ^1^H–^15^N SOFAST-HMQC pulse sequence.

Isotopically labelled [U-98% ^15^N; U-^13^C]-d(TG_4_T), referred to herein as d(TG_4_T), with a purity of over 98% obtained from enzymatic DNA synthesis, was purchased from Silantes (Mering, Germany). Samples were resuspended in Milli-Q water and immediately stored at –80 °C. Different sets of samples were prepared from the water stock: a concentrated solution of 3–4 mM (strand concentration) in 20 mM KCl, in 20 mM NaCl and 90 mM KCl, or in water alone. To obtain a G-quadruplex structure, we followed the common annealing practice by heating at 95 °C for five minutes and chilling at around 2–4 °C in ice-cold water. The cycle was repeated at least two times. The sample in water alone that was used to probe the folding inside cells was also subjected to the annealing process. Vesicular follicle-free *X. laevis* oocytes were prepared according to previous protocols,^25,26^ where 50 nL from a ∼3 mM stock of isotopically labelled [U-98% ^15^N; U-^13^C]-d(TG_4_T) was manually micro-injected into each one of 220–250 oocytes at ∼22 °C. Three different types of sample were produced for micro-injection into *X. laevis* oocytes; d(TG_4_T)_4_ in KCl, K-phosphate buffer (pH ∼ 6.5) (A), in water alone (with a residual amount of NaCl from the supplier) (B), and pre incubated with a 4 times molar excess of ligand 360A^27–29^ under KCl buffer conditions (C). An additional sample was prepared under identical conditions to sample A, and was used to monitor the interaction of previously micro-injected d(TG_4_T)_4_ with a ligand that diffused from the oocytes buffer into the cell interior across the membrane. The damage to each oocyte was assessed under a microscope and the ones showing some minor imperfections from the injection procedure, such as cytoplasmic expansion (micro-bumps), were discarded. Roughly 5–10% of all oocytes had some sort of damage and were discarded. The final intracellular concentration of single-stranded d(TG_4_T)_4_ was close to 100 μM. As a control, some microinjected oocytes were treated with progesterone to test their viability. The oocytes were manipulated and loaded into an NMR tube in modified Barth's saline with 10% Ficoll® (Fluka) buffer, containing ∼120 mM KCl and 20 mM potassium phosphate buffer at pH ∼ 7.2. We decided to place one mL of 20% Ficoll® buffer in the Shigemi™ tube before loading the oocytes, due to its higher density which prevents the bottom oocytes from being crushed over time. The ligand 2,6-*N*,*N*′-(methyl-quinolinio-3-yl)-pyridine dicarboxamide (360A)^27–29^ (see Fig. 1d) was prepared in two different ways. (i) For the passive diffusion experiment, a stock solution in water with 10% DMSO (Fluka) was prepared, of which 5 μL was added to approximately 1 mL of the total volume of the NMR sample solution containing the *X. laevis* oocytes. The final ligand concentration was ∼100 μM. This amount of DMSO (∼0.5%), alone or combined with the ligand, did not induce any effect in control-sets of *X. laevis* oocytes when compared with untreated oocytes (data not shown). (ii) A 2 : 5 molar ratio of ligand and d(TG_4_T)_4_ were pre-annealed.

### NMR experiments

All NMR experiments were executed at approximately 16 °C in order to minimize damage to the cells and at same time improve the observation of the imino signals. The experiments were performed with a 800 MHz Bruker Biospin AG spectrometer equipped with a cryogenic triple-resonance probe, TXI 1H/13C/15N/2H, with Z-gradients. The inclusion of 20% Ficoll® in the buffer and the *X. laevis* oocytes’ robustness, when compared with other cells, allowed us to perform acquisitions for periods longer than 48 h. In order to probe and quantify any possible leakage we replaced the Ficoll® buffer surrounding the oocytes inside the NMR tube with a fresh solution every 12 h, and acquired control spectra to test the leakage from the cells. All 1H-1D spectra were obtained using the 1-1 echo pulse sequence.^30^ Two dimensional (2D) ^1^H–^15^N band-Selective Optimized Flip-Angle Short-Transient Heteronuclear Multiple Quantum Coherence (SOFAST-HMQC) spectra were set with repetition rates of 200–240 ms. We used selective ^1^H pulses centred at 11.2 ppm, covering a bandwidth of 4.0 ppm, with a shape pulse PC9-2.200 as the ^1^H excitation variable flip-angle, and REBURP.1000 (ref. 31) as the refocusing shape pulse. Both *in vitro* and in-cell samples were prepared in 5 mm Shigemi™ tubes supplemented with less than 10% of D_2_O. After reconstitution all samples were left for at least one hour at room temperature before starting spectral acquisition. The 2D spectra were usually acquired with 1024 and 128 complex points in the ^1^H and ^15^N dimensions, respectively, and 32 to 1024 scans were used in different experiments. The SOFAST-HMQC spectra were processed using a cosine-squared apodization function in the time-domain. Equal numbers of real points in the ^15^N dimension were linear-predicted for both in-cell and *in vitro* samples. All spectra were processed and analysed in TopSpin 3.1 (Bruker Biospin AG) and Sparky (T. D. Goddard and D. G. Kneller, SPARKY 3, University of California, San Francisco).

### Peak assignments

The four magnetically-equivalent imino peaks (G2–G5), and nearby low intensity peaks from a second conformer almost imperceptible under KCl conditions, correspond to the d(TG_4_T)_4_ imino signature in the all-parallel four-stranded conformation, with average intensity ratios of the main to the secondary peaks of above five to one. These peaks were previously assigned and are well characterized elsewhere.^12,32^ Additionally, traditional NMR 1D and 2D methods such as HSQC and NOESY spectra were used for ligand titration with 360A.

## Results and discussion

### G-quadruplex d(TG_4_T)_4_ inside *Xenopus* oocytes

**Fig. 2:**
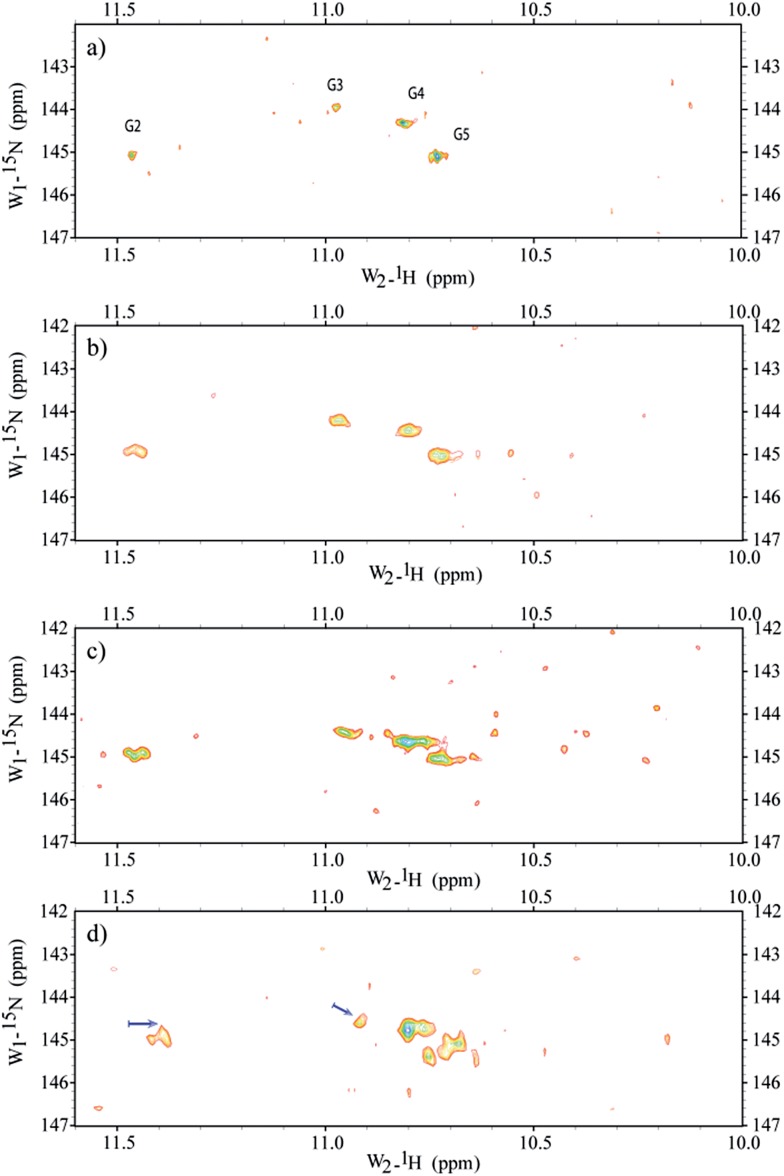
Time spectral evolution of d(TG_4_T)_4_ inside *Xenopus* oocytes. Spectra obtained after a period of 8 h (a), 16 h (b), 26 h (c) and 32 h (d) from assembly of ∼200 oocytes in the NMR tube. The sample buffer surrounding the oocytes inside the NMR tube was replaced every ∼12 h with a fresh solution, and a spectrum of the buffer where the oocytes were resuspended was measured under the same conditions as the control. All controls revealed that no apparent leakage occurred for periods up to 24 h. Between 26 and 32 h some leakage was visible and after 36 h there were several damaged cells and naturally the “leakage” into the surrounding buffer was substantial (data not shown). The results show that d(TG_4_T)_4_ is a robust structure that possesses a good spectral time-window (∼24 h), allowing the study of its interaction with ligands inside *Xenopus laevis* oocytes. Arrows indicate the most pronounced chemical shift deviation (∼0.1 ppm).

Micro-injections of isotopically enriched proteins and nucleic acids were successfully employed in previous studies^17,19,20,25,26,33,34^ with excellent results. Fig. 1a depicts the general organization of a tetrad of guanines that can be found in G-quadruplex structures, where we can visualize the Hoogsteen base-pairing stabilized by the hydrogen bond network and a monovalent cation at the centre. Fig. 1b depicts a stack of four tetrads with four guanines, each lying in a quasi-perfect plane. Fig. 1c shows a typical sample containing ∼200 oocytes inside a 5 mm Shigemi tube. Finally, Fig. 1e shows a characteristic spectrum of the imino region of d(TG_4_T)_4_ in KCl buffer obtained under *in vitro* conditions and recorded using a 2D ^1^H–^15^N SOFAST-HMQC type experiment. Additional experiments were carried out to identify any possible leakage from the oocytes. The sample buffer surrounding the oocytes inside the NMR tube was replaced every ∼12 h with a fresh solution, and spectra of the buffer where the oocytes were resuspended was measured under the same conditions as the control. Parts a–d in Fig. 2 depict the sample time evolution and stability inside the oocytes after approximately 8, 16, 26 and 32 h of spectra acquisition, respectively. In the first spectrum, the folding pattern derived from the in-cell experiments contains four unique peaks, and seems to resemble a unique folding with four tetrads in a parallel fashion, similar to what is commonly found under KCl conditions obtained *in vitro*.^12,35^ No significant line broadening evolution was observed during the period of time of ∼8 h, although it remains higher than what is observed *in vitro* (22–40 Hz *versus* 16–18 Hz (*ω*_1_ – *ω*_2_), respectively). There are no other visible peaks in the NMR spectrum that could indicate different folded species, but as time progress (Fig. 2b and c) new peaks become visible despite the resemblance of the overall resonance-pattern. The new peaks are initially more pronounced for the Gua-2 and Gua-5 tetrads (where Gua-2 and Gua-5 are the guanines at positions 2 and 5 in the single strand respectively), and may represent a new conformer with both ends in conformational exchange, representing a new species in equilibrium with the original more stable one. It can also represent conformers with specific interactions with cellular macromolecules or derived from slow-exchange equilibrium between a mix of G-quadruplex species charged with K^+^ and other abundant ions such as Na^+^. Towards the end of the experiment, as shown in Fig. 2c, the newly formed peaks are more prominent but still have less intensity when compared with the initially observed four major peaks. Moreover, Gua-2 seems to fit three individual peaks instead of a single one. In the bottom panel (Fig. 2d) the spectrum shows that G2 and G3 have shifted by around 0.1 and 0.05 ppm respectively, and additional peaks appear near G4 and G5.

All controls revealed that no apparent leakage occurred for periods up to 24 h. Between 24 and 36 h some leakage was visible and after 36 h there were several damaged cells and the “leakage” into the surrounding buffer was substantial. Therefore, we establish a ∼24 h period as a safe window for the spectral acquisition where we could operate without influence from cellular leakage. Previous NMR^11,36,37^ and crystallographic^38^ experiments have shown that oligonucleotides with the propensity to form G-quadruplexes can adopt a variety of conformers, depending on different parameters such as the sequence and the ionic species stabilizing the tetrads. The typical physiological concentration of monovalent ions, with optimal affinity for G-quadruplexes, inside *X. laevis* oocytes is in the order of 21 mM and 90 mM for Na^+^ and K^+^, respectively,^39^ with a protein concentration of up to 400 g L^–1^.^40^ Having that in mind, we anticipated a more complex spectral pattern for the imino region, as expected for the existence of multiple hybrid conformations in a rather complex cell interior “buffer”. The 2D spectra that we present in this study are well resolved and all four tetrads of d(TG_4_T)_4_ can be individually distinguished. Additionally, no apparent multiple structural conformers are visible from the spectra acquired in the first 16 h post acquisition. The high spectral resolution and lack of extensive line broadening indicates the formation of fast tumbling species on the NMR time scale (μs to ns).^41^ Towards the end of the “safe” spectral window acquisition time, which we set to approximately 24 h, other conformers start to appear, which were observed neither under *in vitro* conditions, with K^+^ or Na^+^, nor in mixtures of both ions (Fig. S1 in the ESI†). Under *in vitro* conditions, once the tetrads are formed they remain stable and unchanged (observed by NMR) for several weeks. This leads us to think that only after a period of ∼24 h do we start to observe substantial binding or degradation of d(TG_4_T)_4_ exogenously administered into the cells. After that period of time, the modifications of d(TG_4_T)_4_ observed inside cells, and without ligand, seem to originate from other macromolecules such as enzymes and co-solutes naturally occurring inside the oocytes, which will with time bind to and change the chemical shift environment felt by the guanines, with additional peak broadening. Nevertheless, these changes are not severe to the point of perturbing the general fold of d(TG_4_T)_4_, thus further attesting to the robustness of this oligonucleotide sequence for tests inside living cells. Further studies may be necessary to probe if the inherent properties of the crowding environment are more important than the specificity of the constituents that make the cell interior so unique, and induce the small folding modification observed after a 16–20 h period spent inside *X. laevis* oocytes. At a low concentration of Na^+^ under *in vitro* conditions, the spectra indicate the formation of a mixture of conformers. When the same sample is microinjected inside the cells, it shows a unique spectral pattern characteristic of a single species, revealing that the oligonucleotide exchanged the Na^+^ ion and refolded inside the cells into a structure pattern identical to the one found in both *in vitro* and in-cell samples pre-incubated under K^+^ conditions (Fig. S1 in the ESI†). This information is relevant to the study of other species more stable under *in vitro* conditions in the presence of Na^+^ when compared with K^+^, such as the human telomeric repeat (22AG). Does this means that all studies *in vitro* should preferably be performed under K^+^ conditions for other G-quadruplexes as well? Other in-cell studies will be necessary, using different G-quadruplex species such as c-myc and 22AG to assert the influence of K^+^ over Na^+^ as the major stabilizing ion within the tetrads. If the K^+^-bound 22AG conformer(s) are the predominant species *in vivo* as well, as our unpublished results and recent studies may indicate,^8^ further studies are necessary to probe the importance of the environment in rational drug design.

### Ligand binding to d(TG_4_T)_4_

**Fig. 3:**
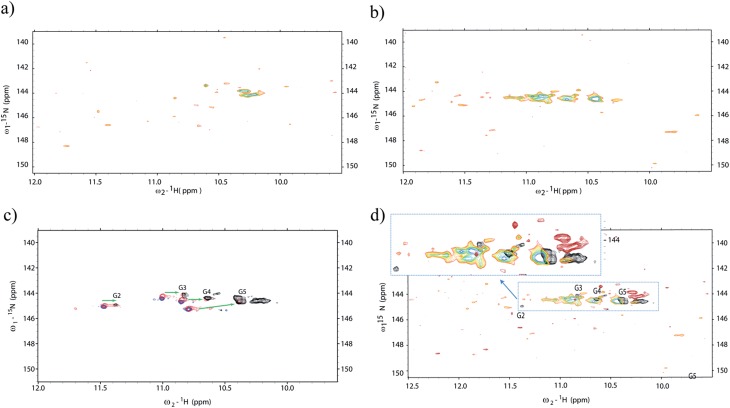
Probing the ligand interaction with d(TG_4_T)_4_ using ^1^H–^15^N SOFAST-HMQC *in vitro* and in-cell. (a) Ligand incubated and freely diffused from the NMR tube buffer to the oocytes interior, previously microinjected with d(TG_4_T)_4_. In (b) we can see the in-cell spectrum that resulted from the incubation of a 2.5 molar ratio of ligand 360A with d(TG_4_T)_4_ for a period of 4 hours (room temperature) prior to co-microinjection in ∼200 *Xenopus* oocytes. Part (c) depicts the NMR titration experiments *in vitro* without ligand (blue), after incubation with 1 (red) and 2.5 (black) molar ratios of ligand to moles of d(TG_4_T)_4_. (d) Shows different spectra from (a) and (b) overlaid with the black spectrum of (c). All spectra were acquired at 16 °C. The spectra clearly show important differences in the organization of the d(TG_4_T)_4_ tetrads after ligand binding *in vitro* compared to the changes observed in-cell. All spectra were analysed taking into account peak assignments described previously^12,32^ together with *in vitro* ^1^H–^15^N HSQC titration of d(TG_4_T)_4_ with 360A.

Additionally, we decided to probe the effects of ligand binding to the micro-injected G-quadruplex structures inside living cells using NMR spectroscopy. To that aim, we selected the ligand 2,6-*N*,*N*′-(methyl-quinolinio-3-yl)-pyridine dicarboxamide (commonly referred to as 360A). Since its discovery in 2004 (ref. 42) it has been the subject of numerous studies. The ligand 360A is now commonly used as a benchmark for both biophysical and cellular assays as this compound was shown to display a potent affinity and selectivity for telomeric G-quadruplex DNA over duplex DNA.^22^ The binding of 360A to tetramolecular quadruplexes was previously probed using FRET melting assays,^43^ and indicated a stabilization of +20 °C of an intramolecular quadruplex at 1 μM concentration of 360A. This indicates a Kd significantly lower than 1 μM, and probably in the 10–100 nM range. However, the determination of an accurate Kd has proven to be difficult, in part due to aggregation and precipitation at high drug/DNA ratios. The attempt to obtain a titration profile inside living cells may present us with new directions to understand ligand–receptor binding inside cells and improve affinity and specificity in structure-based drug design. Two such experiments were performed in two different sets of batches containing ∼200 oocytes each. In the first experiment, the G-quadruplex was micro-injected inside ∼200 oocytes, and later on, the ligand was incorporated inside the NMR tube and left to diffuse freely during an equilibrium period (1 h) (Fig. 3a). In addition, a second experiment started with an *in vitro* incubation between the ligand and the G-quadruplex prior to micro-injection of the mixture (G-quadruplex + ligand) into oocytes (Fig. 3b). Additional experiments were performed under *in vitro* conditions (blue), where d(TG_4_T)_4_ was incubated with molar ratios of 1 (red) and 2.5 (black) of the same ligand (Fig. 3c). The results show that important changes in the chemical shifts and intensities of the imino peaks occur in the presence of 360A. Finally, in Fig. 3d the spectrum obtained *in vitro* with 360A (black) is overlaid on top of two distinct in-cell spectra with the ligand administered by the two different procedures. Important differences can be appreciated between *in vitro* and in-cell experiments. After the incubation of the cells with the ligand (Fig. 3a), or with the alternative method by co-injection, the spectrum shows a dramatic change after approximately seven hours of spectral acquisition, demonstrating that an interaction occurs between the quadruplex and the ligand. We believe that the spectra show the result of a direct interaction between a G-quadruplex-specific ligand and its target within a complex intracellular environment, and we were able to visualize its effect on the imino pattern. Although the peak intensities are severely diminished and a correct interpretation is rather difficult, the pattern of the correlation peaks shows important differences to what is observed both without the ligand *in vivo* and with the ligand under *in vitro* conditions. Another, but less likely, explanation could be related to an increased degradation of the oligonucleotide once exposed to the ligand. Normally, we can see the imino pattern of newly microinjected d(TG_4_T)_4_ inside *Xenopus* oocytes in less than 4 h, but in the presence of the ligand this was never possible in such short periods of time. Nevertheless, it is encouraging to observe such dramatic changes in living cells, even if a complete ligand titration was not possible. We observe that *in vitro* the ligand induces a pronounced effect on the imino pattern, especially by reorganizing the G2 and G3 tetrads, since their relative peak intensities were significantly diminished and shifted to high field. We also observe the appearance of amino bonds-pattern in the spectra at ∼10.2 ppm, indicating some sort of stabilization induced by the ligand in the amino groups that are normally exposed, and in fast exchange with bulk water. This observation is also present in the *in vitro* spectra. Under *in vitro* conditions all four tetrads appear to remain preserved, albeit with important differences in chemical shift induced by the ligand.

In contrast, the in-cell results indicate two distinct behaviours. In the first experiment, the spectrum shows the lack of any tetrad-signature (Fig. 3a). Surprisingly, the only visible peaks around 10.2 ppm indicate the formation of characteristic amino bonds often seen associated with tetrads. When an excess of ligand is pre-incubated with d(TG_4_T)_4_ before microinjection into the oocytes, the modifications in the G-quadruplex structure occur *in vitro*, and the peak pattern observed for the complex once inside the cells (Fig. 3b) is to some extent similar to what is observed *in vitro* (Fig. 3c, black spectrum). There are small differences that are difficult to evaluate; the peaks are broader and we can see newly formed peaks that were not observed in the experiments without the ligand inside the oocytes, nor under *in vitro* conditions with equivalent amounts of ligand (Fig. 3c). Unfortunately we cannot use longer acquisition periods to give a better signal because that leads to sample degradation under *in vivo* conditions (Fig. 2). A possible explanation is that the ligand is able to stabilize the complex and other biomolecules such as enzymes may interact better with the complex. We cannot exclude d(TG_4_T)_4_ dimerization or intracellular precipitation as an origin for new peaks or peak broadening in the complex formed with 360A. Again, the test for leakage was performed and the results were negative as seen before (Fig. S3 in the ESI†). After a period of ∼20 h, the signals from both experiments totally vanish. We decided to probe if the oligonucleotide–ligand complex was degraded, or if it was not visible due to the interaction with large intracellular molecules. For that, we homogenized the oocytes under cold conditions (∼4 °C) and separated the pellet from the cytoplasm of both samples by centrifugation. Then we measured the spectrum of the cytoplasm under an identical experimental setup. Under these conditions, we did not observe any peaks in either sample preparations with the ligand. However, if we heat the samples at 80 °C to denature proteins that may be bound to the d(TG_4_T)_4_–360A complex, relatively strong peaks appear in the spectrum for the sample pre-incubated with ligand. The peak pattern is somehow different to what is found *in vitro*, but this time we can clearly see what look like three distinct imino peaks (blue spectrum in Fig. S4 ESI†), indicating that at least a part of the complex was not digested by cellular enzymes when the oocytes where intact, and neither was it destroyed by heating at 80 °C. For the sample where the ligand was freely diffused, we did not observe any signal in the imino region before or after heating, which can be related to the fact that during the unfolding procedure at 80 °C we may accelerate the degradation of the oligonucleotide.

## Conclusions

Our results clearly show that G-quadruplex structures form within cells under physiological conditions and are ‘visible’ by NMR spectroscopy. The G-quadruplex made of d(TG_4_T)_4_ seems to be resistant to numerous enzymatic activities and can be considered as a long-lived species within cells. The differences found between both *in vivo* and *in vitro* results raises new pertinent questions that must be addressed in future experiments concerning G-quadruplex ligand interactions. The ligand bound to the oligonucleotide seems to enhance the binding of certain biomolecules or/and precipitates the complex inside the cell which makes it “invisible” on the NMR time scale. A more vast work including in-cell and molecular crowding conditions needs to be carried out to fully understand if ligand binding studies under *in vitro* conditions are pertinent. Taking our findings together with those of previous NMR studies obtained with the human telomeric sequence d(AG_3_(TTAGGG)_3_),^8^ we face newly pertinent information that gives us fresh clues on how to proceed to study oligonucleotides, and more specifically G-quadruplex structures inside living cells with ligands. We expect the findings of this work to encourage further new studies in the recent field of in cell structural biology.
