# Supplementary material for: G-quadruplex DNA and ligand interaction in living cells using NMR spectroscopy
Source: Chem Sci. 2015 Jan 14;6(6):3314–20. doi: 10.1039/c4sc03853c (PMC5490339; doi:10.1039/c4sc03853c)
Supplement: Supplementary file 1 [file SC-006-C4SC03853C-s001.pdf]

Electronic Supplementary Material (ESI) for Chemical Science.
This journal is © The Royal Society of Chemistry 2015

Supporting Information

G-quadruplex DNA and ligand interaction in living cells using NMR spectroscopy.

Gilmar F. Salgado [a,b]*, Christian Cazenave [c,d], Abdelaziz Kerkour [a,b] & Jean-Louis Mergny [a,b]

[a] Univ. Bordeaux, ARNA laboratory, F-33000 Bordeaux, France.
[b] INSERM, U869, IECB, F-33600 Pessac, France.
[c] CNRS, Microbiologie Fondamentale et Pathogénicité, UMR 5234, F-33000 Bordeaux, France
[d] Univ. Bordeaux, Microbiologie Fondamentale et Pathogénicité, UMR 5234, F-33000 Bordeaux, France.
*: to whom correspondence should be addressed: Fax: (+ 33) 5 40 00 30 04;
E-mail: g.salgado@iecb.u-bordeaux.fr or gfjsalgado@gmail.com

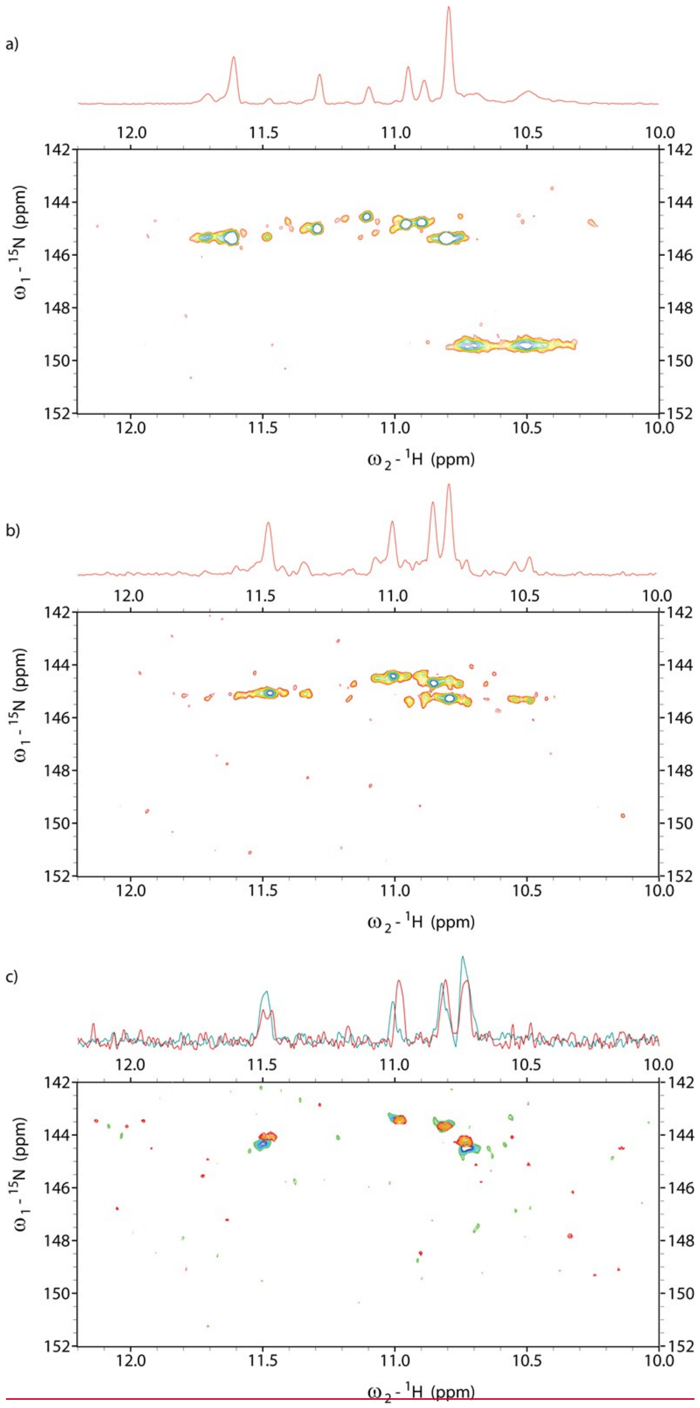

Figure S1. Depiction of a series of ^{1}H-^{15}N SOFAST-HMQC spectra. In part (a) the oligonucleotide d(TG_4T)$_4$ was resuspended in water as received from the supplier, while in sample (b) we included 20 mM KCl (b), and finally inside oocytes after micro-injections of both preparations in two different experimental sets (c). The presence of residual Na^+ ions issued from the solid phase synthesis is perceived from the spectra in part (a), as it shows the existence of multiple imino peaks originating from folded tetrads in different conformations. Sample shown in top panel was subjected to two cycles of warming (95 °C) and cooling (4 °C). In part b of the same figure we can observe the spectra from the sample reconstituted in buffer A (containing KCl) and also treated with two cycles of warming (95 °C) and cooling (4 °C) in order to facilitate quadruplex formation. In panel b and c we can observe four major resonances with similar intensities, arising from each tetrad group of four magnetically-equivalent imino peaks (G2-G5), and smaller peaks nearby from a second conformer. Despite the different d(TG_4T)$_4$ conformations states before the microinjections, only the KCl species seems visible in the NMR time scale inside *Xenopus* oocytes (c). From the 1D projections on the top of each panel we can appreciate the linewidth broadening in the *in cell experiment.* Approximately 1.5-1.7x10^{-8} moles of oligonucleotide was used in each experiment.

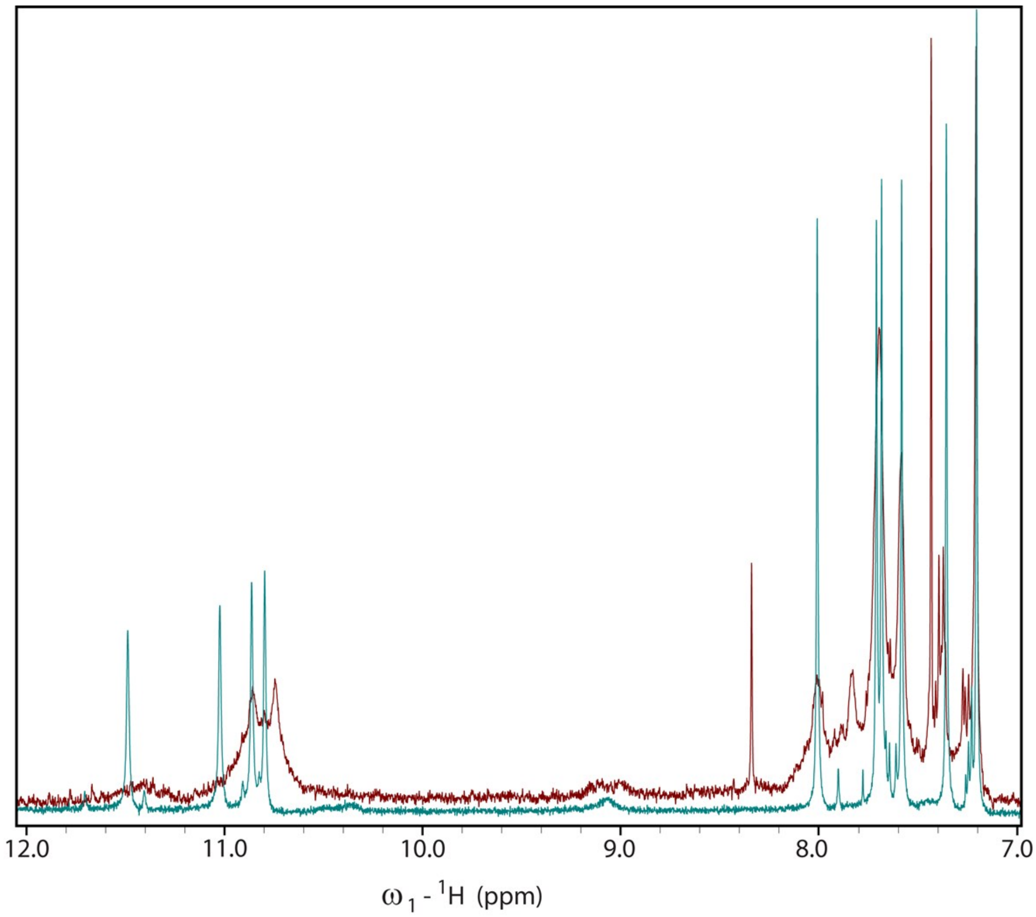

Figure S2. Spectrum of 200 µM of ^{1}H- $d(TG_4T)_4$ in 90 mM KCl evidencing a single species G-quadruplex (green). In red spectrum we can observe that multiple species are present in a buffer mixture containing ~25 mM NaCl and 90 mM KCl. Both samples were treated with two cycles of warming (95 °C) and cooling (4 °C).

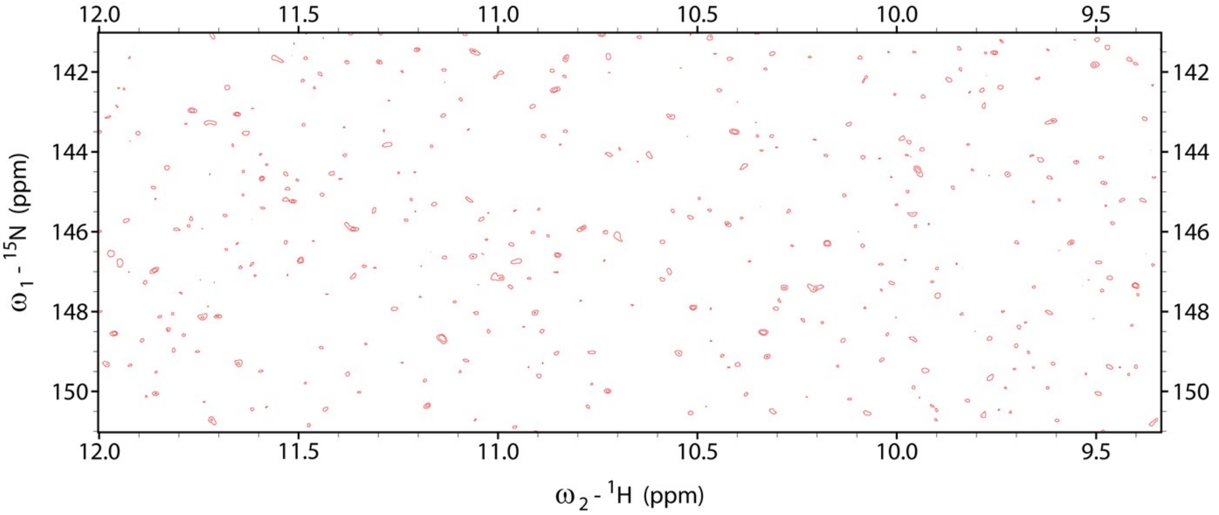

Figure S3. The figure depicts a typical oocyte-buffer control spectrum. We can observe that no imino peaks are observed meaning that no leakage was observed from inside the *X.* oocytes to the buffer outside the cells during the NMR spectral acquisition. This specific spectrum corresponds to the buffer collected after the experiment observed in Fig. 2b.

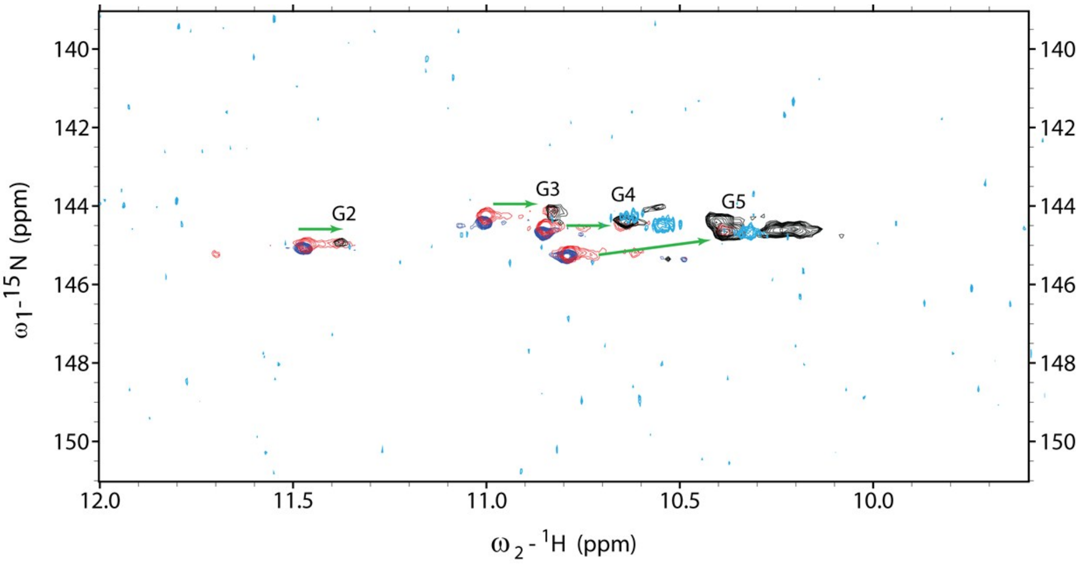

Figure S4. Probing the G-quadruplex-ligand complex in *X.* oocytes cytoplasm extracts. After ~20 h of spectral acquisition the signal from the d(TG$_4$T)$_4$-360A complex inside the oocytes disappears. To probe if the complex was bound to proteins that tumble outside of the NMR time window (nanoseconds to microseconds by solution NMR) or "precipitate" inside the cells we isolated the cytoplasm under cold conditions (~4°C) from ~200 oocytes and acquire a 2D SOFAST spectrum under identical conditions. The result showed no visible peaks. Finally the same cytoplasmic preparation was heated for 2 minutes at 80 °C and a spectrum collected under same conditions (light blue), overlaid on top of spectra from Fig. 3c. The appearance of three distinct imino peaks evidences de binding of the d(TG$_4$T)$_4$-360A complex to proteins that were denatured by heating the sample at 80 °C. The spectrum shown in light blue is only for sample of d(TG$_4$T)$_4$-360A that was pre-incubated previous to micro injection. To the second sample, where the ligand diffused freely inside the oocytes we did not observed any signal before and after heating the supernatant.

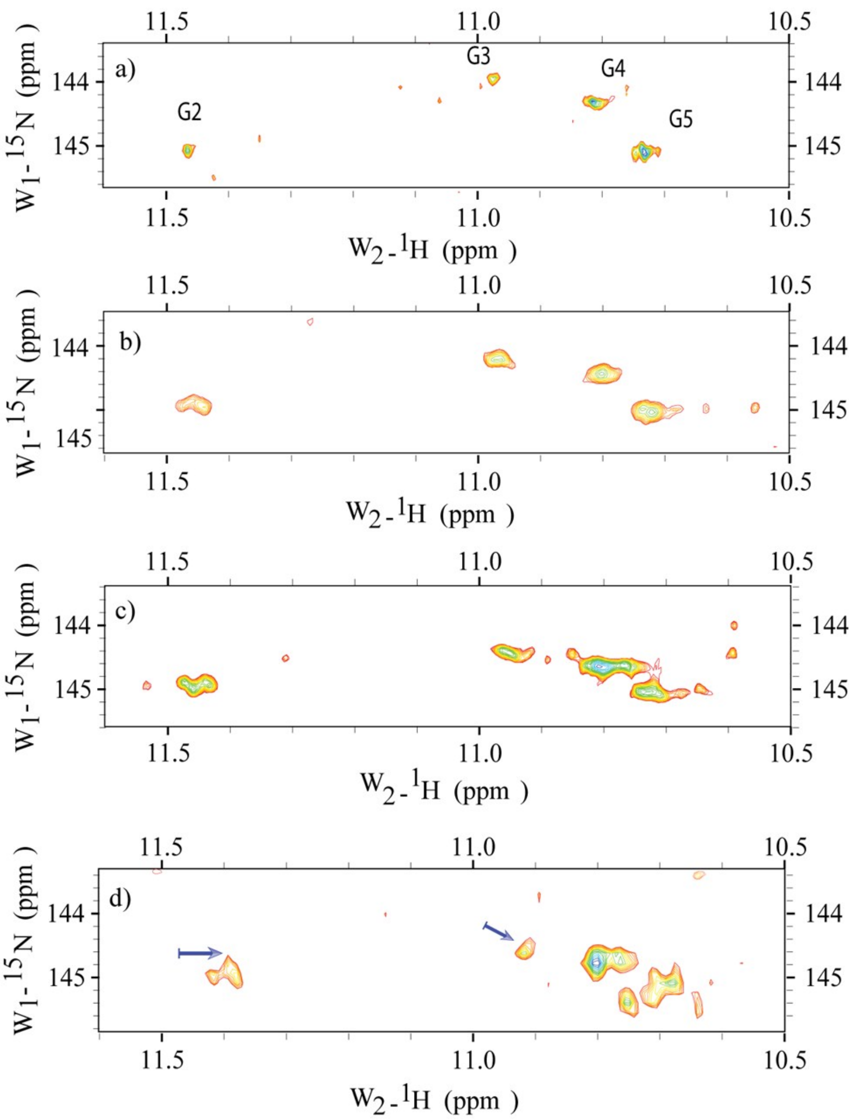

Figure S5. Magnified version of figure 2 from main text.
